# Seedling Emergence from Seed Banks in *Ludwigia hexapetala*-Invaded Wetlands: Implications for Restoration

**DOI:** 10.3390/plants8110451

**Published:** 2019-10-25

**Authors:** Brenda J. Grewell, Morgane B. Gillard, Caryn J. Futrell, Jesús M. Castillo

**Affiliations:** 1 USDA-ARS Invasive Species and Pollinator Health Research Unit, Department of Plant Sciences MS-4, University of California, Davis, 1 Shields Avenue, Davis, CA 95616, USA; 2 Departamento de Biología Vegetal y Ecología. Universidad de Sevilla, Ap. 1095, 41080 Sevilla, Spain

**Keywords:** invasion ecology, invasive species, plant invasions, wetland restoration

## Abstract

Soil seed banks play a critical role in the maintenance of wetland plant communities and contribute to revegetation following disturbances. Analysis of the seed bank can therefore inform restoration planning and management. Emergence from seed banks may vary in response to hydrologic conditions and sediment disturbances. To assess the community-level impact of exotic *Ludwigia hexapetala* on soil seed banks, we compared differences in species composition of standing vegetation among invaded and non-invaded wetlands and the degree of similarity between vegetation and soil seed banks in northern California. To determine potential seed bank recruitment of *L. hexapetala* and associated plant species, we conducted a seedling emergence assay in response to inundation regime (drawdown vs. flooded) and sediment depth (surface vs. buried). Plant species richness, evenness, and Shannon’s H’ diversity were substantially lower in standing vegetation at *L. hexapetala* invaded sites as compared to non-invaded sites. Over 12 months, 69 plant taxa germinated from the seed banks, including *L. hexapetala* and several other exotic taxa. Seedling density varied among sites, being the highest (10,500 seedlings m^−2^) in surface sediments from non-invaded sites subjected to drawdown treatments. These results signal the need for invasive plant management strategies to deplete undesirable seed banks for restoration success.

## 1. Introduction

Exotic plant invasions are a major threat to native ecological communities and can significantly reduce native species diversity [1]. The management of invasive plants has become a conservation priority that is an essential component of wetland ecosystem restoration. While weed management actions that remove dominant invaders might be expected to promote native species through competitive release, this disturbance can promote secondary invaders and lead to an increase in undesirable exotic species [2,3,4]. While the potential role of seed banks in both secondary invasions and restoration has long been recognized [5,6], very few scientific studies or restoration projects have evaluated the relationships between weed invasions and soil seed banks, despite their important role in vegetation dynamics [7]. Therefore, these ecological relationships are rarely considered in ecological restoration plans.

Recognition of the importance of soil seed banks to the maintenance of wetland plant communities was first reported by Darwin, from observations of plant emergence from soil collected at the edge of a pond [8]. Colonization and recruitment of invasive plants have become significant challenges to the restoration and management of wetland conservation areas. Riverine wetlands and riparian zones experience dynamic hydrology and regular disturbances that create opportunities for the recruitment of invasive alien plants [9,10]. These wetlands are highly prone to invasion due to hydrologic connectivity that facilitates the rapid hydrochorous dispersal of invasive plant propagules throughout watersheds [11,12,13]. Re-establishment of native emergent wetland plant communities is particularly important, given the vital roles of rooted aquatic macrophytes in the structure and functioning of shallow freshwater ecosystems [14] and in providing ecosystem services such as floodwater retention and improvement of water quality [15]. Significant obstacles to wetland restoration success are unexpected developments that can result from plant invasions, their seed dispersal, and seed bank dynamics [15,16]. Most efforts to investigate the impacts of invasive plants on native plant diversity have compared the standing vegetation in invaded and uninvaded communities, yet the emergent vegetation is not the only component of overall plant community diversity [7]. The impact of plant invaders on seed banks can be quite different from their impacts on above-ground vegetation [17,18,19]. While there has been a paucity of studies directly assessing the impact of plant invasions on soil seed banks, recent metadata analyses of available data indicate significant decreases in native species richness and native seed bank density in seed banks at sites where invasive plants were present in standing vegetation, and there were no cases of increases in native seed bank richness at invaded sites [7] The identity of the invasive species played a role, with some invaders having greater negative impacts than others, and riparian and coastal wetlands were among the most impacted habitats [7]. Previous investigations have documented post-invasion changes in standing vegetation as well as seed bank composition-impoverished communities dominated by weedy species, and as invasions proceed, the changes in standing vegetation will increasingly impact the soil seed bank [7,18].

A comparison of standing vegetation and soil seed bank life stages at invaded and uninvaded sites can clarify invasion impacts and provide valuable information for conservation management [20,21,22]. Seed banks formed from invasive plants will affect their response to temporal variations in novel environmental conditions. Soil seed banks formed from native species will also affect their response to changing environmental conditions, including changes that may result from the introduction of an invasive plant species [23]. Soil seed banks often play a significant role in plant community assembly and restoration [9,23], as they represent past and/or present species presence and propagule pressure as well as potential future plant communities. The evaluation of seed bank composition is therefore useful for detecting both rare and invasive plant species that may have a cryptic presence as buried seeds [24,25]. A high degree of similarity between standing vegetation and species pools stored in seed banks has been observed in some freshwater wetlands [26,27], while others are dissimilar [28,29,30]. While these relationships vary by site, analysis of the seed bank can detect problematic species that could hinder the restoration of wetland communities [31]. The importance of the regenerative potential of seed banks is magnified following disturbances imposed for weed management. A better understanding of the potential role of seed banks on future vegetation dynamics in invaded and uninvaded habitat can provide critical information to improve restoration planning and integrated weed management strategies. This is especially important in a context of global and local changing environments. For example, increases in environmental temperatures can increase the germination capacity of aquatic plant species such as *Ludwigia hexapetala*, suggesting the need for long-term management to address continued secondary invasions from seed bank emergence [32]. Invasive plants and climate warming both represent strong mechanistic forces that can change local abiotic conditions and thereby affect seed germination, viability, and dormancy [23,32] This points to the need for further evaluation of the impact of interacting environmental changes, including plant invasions and climate warming, on soil seed banks and vegetation dynamics [23].

Within aquatic ecosystems, the recruitment of plants from the seed bank can be regulated by both hydrological and sediment deposition processes [33]. Variation in water depth consistently has been shown to affect the establishment of hydrophytes from seeds [34,35,36,37]. Submersed aquatic plants typically germinate in flooded conditions, though low oxygen and light levels under water may decrease seed dormancy loss and prolong seed bank longevity [38]. However, seeds of many emergent species do not germinate until oxygen and temperature conditions improve after flood water recedes [28,34]. In floodplain habitats with pulse inputs of water, variable patterns of inundation and exposure of drawdown zones explain the recruitment of vegetation from seed banks [36,39]. Furthermore, differential sedimentation patterns in a given watershed directly affect the depth distribution of soil and seed burial and can reduce seed germination [40,41]. Therefore, the disturbance of sediment profiles imposed by restoration activities may alter seed exposure to varying degrees, depending on the location within a watershed.

In this context, the analysis of seed bank composition and emergence response to contrasting environmental conditions can greatly inform wetland restoration planning. The seed bank may be a useful indicator of the presence of potentially aggressive invasive weeds, informing the likelihood of success in restoring desirable vegetation [42,43,44]. If desirable species of the community are either absent or unable to be recruited from the seed bank under degraded environmental conditions imposed by invasion, the contribution of the seed bank to community restructuring may be limited [45]. The identification of a persistent seed bank of invasive weed species can also help prioritize limited resources towards the management of species with potentially large impacts [46] and may indicate the effort required for successful control. While management typically targets the suppression of a particularly dominant invasive weed, consideration of seed bank dynamics may lead to focusing resources on preventing secondary invasion of otherwise unanticipated nuisance weeds that could hinder the re-establishment of desirable plant communities.

Here, we examine the impact of an invasive aquatic weed, *L. hexapetala* (Hook. & Arn.) Zardini, H. Y. Gu & P. H. Raven (Uruguayan primrose-willow) on emergent wetland vegetation and associated seed banks in a flood-pulse wetland complex in the Laguna de Santa Rosa of northern California (North America). *L. hexapetala*, a plant species of South American origin, has been naturalized in California for at least 75 years [47]. Over the past two decades, it has become an aggressive invader of riverine and palustrine wetlands in the Sacramento Valley and coastal regions of California [48], spreading throughout the Russian River Watershed including the backwater floodplain channels of the Laguna de Santa Rosa where infestations are severe. We investigated seed bank factors relative to *L. hexapetala* invasion that can influence weed management and wetland restoration efforts. We addressed the following objectives: (1) Compare emergent vegetation in invaded and uninvaded field sites to describe associated changes in plant species composition and diversity in invaded communities; (2) Evaluate the effects of invasion status, inundation, seed bank burial depth and their interactions on the emergence, diversity, and provenance of seedlings from experimental seed banks; and (3) Assess the similarity between the seed bank community and the extant vegetation in invaded and uninvaded wetland sites. On the basis of previous research, we hypothesized that (1) The extant above-ground vegetation would not reflect plant community composition in the seed bank; and (2) Both increased seed burial and flooding would reduce taxa density and emergence of seedlings from seed banks.

## 2. Results

### 2.1. Invader Impact on Community Composition and Diversity

**Figure 1:**
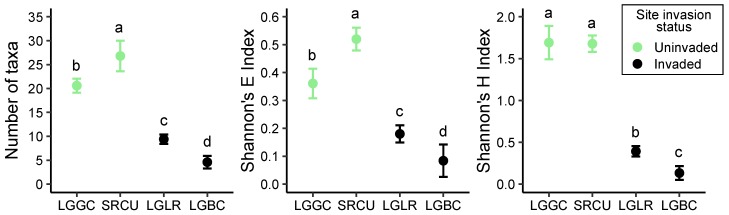
The effects of *Ludwigia hexapetala* (LUHE) invasion on biodiversity components of the plant community (mean ± SE) including species richness (number of taxa), evenness (Shannon’s *E*), and diversity (Shannon’s H’) at Laguna at Gravenstein Creek (LGGC), Santa Rosa Creek above Laguna (SRCU), Laguna at Laguna Ranch (LGLR), and Laguna at Blucher Creek (LGBC). Data points represent means with standard error bars.

The presence of the invader *L. hexapetala* was associated with pronounced decreases in plant species richness, evenness, and diversity in the Laguna de Santa Rosa, though the magnitude of these effects varied by site (Figure 1). At nearby uninvaded sites, species were more evenly distributed, and richness was higher at an upstream tributary marsh (SRCU) than in the central reach of the Laguna (LGGC), but overall diversity was comparable. The abundance of *L. hexapetala* at invaded sites was not statistically different (paired t-test, Bonferroni adjusted *p* = 0.131), yet all aspects of plant species diversity were the lowest at LGBC which was heavily invaded (Figure 1).

### 2.2. Seeding Emergence Assay

**Figure 2:**
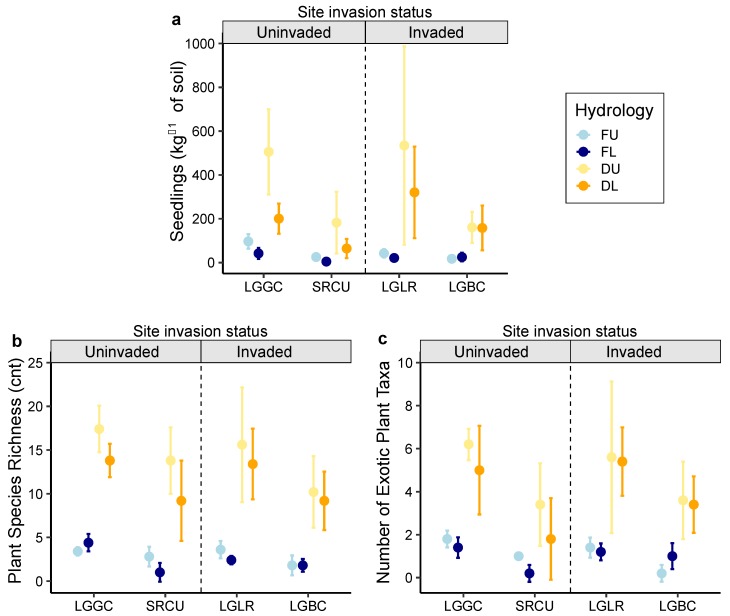
Effects of hydrology treatment (F = flooded, D = drawdown, moist soil), depth of soil profile (U = upper/surface; L = lower/buried), and wetland location on **a**) total seedling emergence density (seedlings kg^−1^ of soil), **b**) plant species richness, and **c**) exotic plant taxa richness in seed banks from the Laguna de Santa Rosa. Site and species codes are defined in Figure 1. Data points represent means with 95% confidence interval error bars. Some error bars are too small to be visible.

We recorded 4075 emergent seedlings from sampled seed banks, including 69 plant taxa from 28 families (Table S1). The total numbers of seedlings and species richness were the highest from the surface soil layer that was not flooded but had constant moist soil conditions, and emergence varied by site (Table 1, Figure 2; Table S2). The interactions between hydrology and burial depth did not influence either seedling emergence or taxa richness (Table 1). The response to hydrology and sediment depth was similar at invaded and uninvaded sites, where *L. hexapetala* was absent in standing vegetation. In each case, regardless of the invasion status, the hydrologic conditions had greater influence on the recruitment from the seed bank than soil burial. A significant number of exotic plant seedlings (1334) emerged from the seed banks, including several taxa known to be invasive in wetland communities (Table S1). Overall, 33% of all seedlings (3377 exotic seedlings kg^−1^ of soil sample) and 29% of plant species that were recruited from seed banks were exotic invasive weeds. Exotic seedling density was the greatest from surface soils under non-flooded, moist soil conditions (Table 1, Figure 2, Table S2).

Exotic taxa density was significantly affected by the interaction between site and soil depth (Table 1). The most abundant exotic recruits were *Alisma lanceolatum* With., *Mentha pulegium* L., *Crypsis schoenoides* (L.) Lam., *Lythrum hyssopifolia* L., *Agrostis stolonifera* L. and *L. hexapetala*. While not as abundant, the recruitment of additional weed species known to be management problems included *Lepidium latifolium* L., *Ludwigia peploides* (Kunth) P.H. Raven ssp. *montevidensis* (Spreng.) P.H.Raven, *Lythrum salicaria* L., *Phalaris aquatic* L. and *Phalaris arundinaceae* L. *L. hexapetala* seedlings emerged from both surface and buried soil layers at both invaded sites that were subjected to non-flooded conditions. No *L. hexapetala* seedlings emerged from any flooded treatment, or from non-invaded sites.

In general, flooded treatments limited the total numbers of seedlings, the taxa richness of the emergent community, and the number of exotic seedlings (Figure 2). However, exotic *A. lanceolatum* and *L. hyssopifolia* germinated in high numbers while inundated. At sites where *L. hexapetala* had invaded, the emergence of seedling from inundated, buried seed banks was depressed compared to uninvaded marshes. Num DF refers to numerator degrees of freedom, and den DF refers to denominator degrees of freedom for the F test statistic; * indicates interaction of effect variables.

Soil physico-chemical properties associated with seed bank samples varied among sites and with profile depth (Figure S3). Descriptive statistics were performed on these data. A qualitative comparison of mean values indicated soil organic matter, total soil carbon (C), nitrogen (N), and phosphorus (P) content were all higher at lower watershed sites (LGGC, LGLR) than at the more upstream sites (SRCU. LGBC), reflecting the influence of watershed position rather than *L. hexapetala* invasion status. Total soil P levels (from 0.4 to >0.5 mg P g^−1^ DW) indicated eutrophic conditions at all study sites. However, soil N/P ratios were more elevated at the more downstream sites, from which the highest numbers of exotic taxa emerged from seed banks collected from both surface and buried soil layers (Figure 2).

### 2.3. Comparison of the Soil Seed Bank and Extant Vegetation

**Figure 3:**
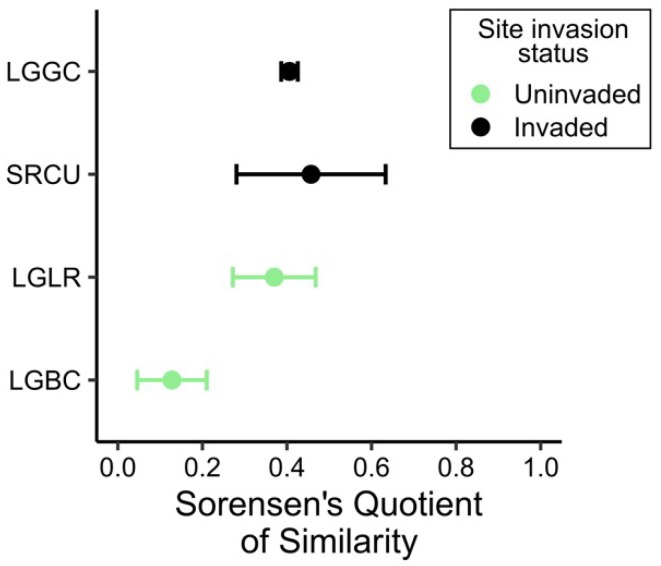
Sørensen’s quotient of similarity comparing standing vegetation and seed banks for *L. hexapetala*-invaded and uninvaded marshes in the Laguna de Santa Rosa. Sørensen’s quotient = 1 indicates complete similarity. Site and species codes are defined in Figure 1. Data points represent means with 95% confidence interval bars.

A total of 87 plant taxa were recorded among extant vegetation and seed banks assayed across all study sites, including 32 monocots and 55 dicots, and of these, 49 plant taxa were common to extant vegetation and seed banks (Table S1). Sixty-six plant taxa were observed in extant vegetation, which is comparable to the 69 taxa observed in seed banks, yet the floristic compositions of the two were quite different (Figure 3). In general, seed bank species pools did not closely resemble standing vegetation. *L. hexapetala* invasion decreased the similarity between extant vegetation and seed banks, as Sørenson’s similarity indices were the highest at uninvaded sites (Figure 3). The effect of the invasion on this similarity was most pronounced at LGBC, where *L. hexapetala* abundance was the highest. There was a significant propagule bank at the invaded sites that was not reflected in standing vegetation (Table S1).

## 3. Discussion

The natural recruitment of plant species after disturbances that open seed germination niches (e.g., weed management activities, flood disturbance) reflects the potential contribution of buried seed banks [49]. Seed bank recruitment of desirable native plant species following disturbances can contribute to the recovery and regeneration of vegetation, but the emergence of cryptic invasive species from soil seed banks can drive vegetation succession and compromise the restoration goals. Our results indicate that the Laguna de Santa Rosa wetlands maintain a large and diverse soil seed bank, of which nearly one-third is composed of exotic weed species known to be invasive in wetland ecosystems. The presence of exotic species in soil seed banks, including the dominance of some, is a common finding in highly disturbed sites [50,51] and gives rise to secondary invasions [7,52,53]. The presence of invasive species in the seed bank indicates secondary invasions should be expected during restoration and suggests the need for vigilant monitoring and rapid comprehensive weed management approaches.

Among the exotic species pool at our study sites were viable seeds representing a persistent life stage of the aggressive invader *L. hexapetala*. This species appears to be well adapted to pulse-flood watershed conditions, for it can take advantage of both flood and drought cycles due to its efficient hydrochorous dispersal and colonization by vegetative fragments [13,54], flotation ability of seed capsules, sexual reproduction ability [55], and high germination capacity [32,56]. In addition, as demonstrated in this study, *L. hexapetala* was able to maintain viable seed banks and recruit from seed banks in moist soil that is exposed following a flood pulse recession.

Although wetland vegetation is often maintained by the vegetative expansion of dominant species, moderate disturbance can create gaps and trigger the germination of seeds present in the soil. Opportunities for seed bank recruitment are expected to increase following disturbance imposed for weed eradication and active wetland restoration efforts. While it is difficult to predict the rate and direction of plant community development during early succession, colonization of vegetation [57] and development of seed banks [58] can be rapid during the restoration of freshwater wetlands. In this study, the seed bank composition was floristically quite different from that of the standing vegetation, with the most dramatic impact of the invasion documented at the LGBC study site. Given these below-ground vs. above-ground differences in plant species composition, shifts in community composition following disturbance should be expected [59]. Our results are consistent with other reported findings of low plant species similarity between soil seed banks and extant vegetation across a range of ecosystems (see review, [60]).

While similarity analyses suggest seed bank composition is not the primary driver of vegetation within study plots in the Laguna de Santa Rosa, disturbance could prompt a shift that increases the relative contribution of seed bank recruitment. Our results provide previously unknown details on the composition of native and exotic plant species pools residing in soil seed banks that are expected to contribute to revegetation following disturbance. Knowledge of this cryptic ecosystem component provides an opportunity to adapt weed management strategies for the primary invader, *L. hexapetala*, as well as to prepare and manage for a suite of secondary invaders that are present in the seed bank and will likely germinate and emerge as future problems under particular environmental conditions.

While standing vegetation in wetlands is often described and monitored over time, little attention is paid to the seed bank, which in many cases can be the most long-lived life stage of plant species and can influence community succession. The seedling emergence method to assess seed banks is used over short timescales and may therefore underestimate the size and species richness of the seed bank [61]. However, in the context of restoration of an invaded plant community, results such as those reported here can provide important predictive information regarding seed bank responses to potential restoration actions. Seedling emergence from experimental seed banks also provides knowledge of viable seeds, and the seedlings that emerge are easier to identify than the seeds extracted from soil [62]. The seed bank represents a pool of regenerative potential that is already present at a given site, and recruitment from seed banks is one example of the impact of weeds that can persist long after the removal of their standing biomass. Because the successful control of invasive exotic plants largely depends on the regenerative potential of the target invasive weed and other plant species that might also be influenced by removal efforts [63], sustainable weed management must consider both seed bank and extant vegetation responses to control options.

Because *L. hexapetala* establishes persistent seed banks, its management should also target the removal of biomass prior to the filling of seed capsules to limit new seed dispersal. Our results, showing invasive *L. hexapetala* seedling emergence from both the surface and the deeper soil from invaded sites, suggest that short-term weed control actions will likely be ineffective, and managers must adopt long-term strategies. Sediment removal can be a successful strategy for the removal of weedy exotic species from the seed bank [64], but this approach also removes native propagule banks that encompass the evolutionary history of the community and can include rare species.

Predicted global temperature and drought increases due to climate change are expected to result in higher water temperatures, which could result in greater areas of drawdown and reduced flooded areas within perennial wetlands. If these effects are manifested, our results predict an increased role of seed bank recruitment within wetland communities, including the recruitment of more exotic weed species. In contrast, predicted increases in soil temperature are another anticipated outcome of global climate change, and empirical evidence suggests that seed bank persistence will decrease with increasing soil temperatures [55,56,65], though both native and exotic species will likely be impacted. In some cases, the restoration managers may be able to manipulate hydrology to expose moist sediments to higher temperatures and thereby accelerate the depletion of weed seed banks.

The details revealed by this experimental study provide key insight of the predictive power of seed bank assays for sustainable weed management strategies that are applicable for a range of restoration efforts in invaded ecosystems. This example reinforces the need for active partnerships between scientists and restoration managers [66], as results from experimental studies can help prioritize control efforts, contribute to improved understanding of sources of variability in plant community development, and refine holistic approaches to the restoration of weed-impacted conservation lands. The knowledge of seed bank responses to environmental conditions gained from this study has important implications for the development of strategies to enhance the conservation status of degraded wetland ecosystems invaded by *L. hexapetala*. These findings improve our ability to predict future contributions of stored seed pools to native vegetation succession and thereby provide an opportunity to tailor pro-active management strategies relevant to the ecological characteristics of both desirable and nuisance plants.

## 4. Materials and Methods

### 4.1. Study Area and Focal Invasive Species

**Figure 4:**
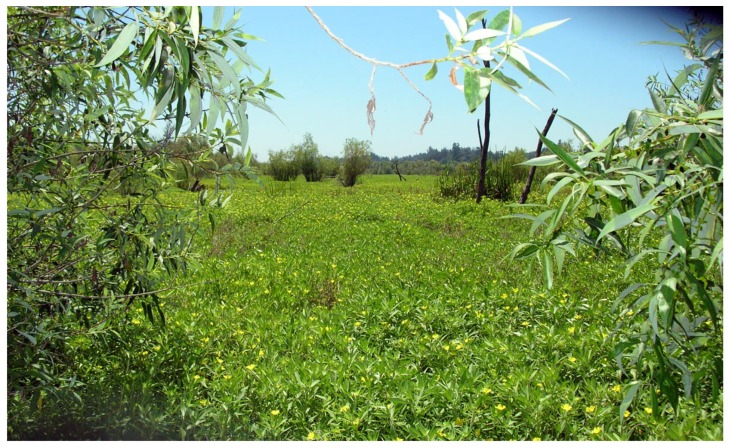
Photograph of *Ludwigia hexapetala* invasion in Laguna de Santa Rosa wetlands (Sonoma County, CA, USA).

This study was conducted in the Laguna de Santa Rosa sub-basin of the Russian River Watershed (centerpoint: lat 38°26′ N, long 122°43′ W), approximately 90 km northwest of San Francisco, California, USA. The freshwater wetland complex of the Laguna de Santa Rosa is designated a wetland of international significance by the Ramsar Convention [67]. Historically, the complex included significant areas of wetland and riparian habitat, of which approximately 67% has been lost primarily due to agricultural land conversion. Restoration of lost or degraded wetlands and ecological functions of this unique freshwater ecosystem within the coastal region of California is a conservation priority [68]. The Laguna de Santa Rosa channel merges with Mark West Creek to form the largest tributary to the Russian River. During winter storm pulses, the Russian River supplements local watershed runoff as it backflows into the Laguna, inundates the broad floodplain, deposits sediment, and then recedes. The U.S. Environmental Protection Agency lists the Laguna channel as an impaired water body due to water temperature, N, P, mercury, dissolved oxygen, indicator bacteria, and sediment levels [68]. Over the past decade, the aquatic weed *L. hexapetala* has spread and flourished in the degraded conditions of the Laguna, where it has challenged watershed goals for the restoration of desirable biological communities and ecosystem processes [46] (Figure 4).

Aquatic *Ludwigia* taxa are among the 200 most aggressive world invaders [69]. They are the most significant nuisance weeds in French rivers [70]. The species tolerates a broad range of ecological and climatic conditions [70,71]. The aquatic *Ludwigia* species are emergent perennial herbs that are rooted in the substrate and have long prostrate or ascending shoots that root and branch at stem nodes and creep across the water surface to form dense tangled floating mats [72]. In areas where water recedes from the surface during dry periods, *L. hexapetala* can survive desiccation and produce ascending shoots that can exceed two meters in height. Seeds are embedded in the woody endocarp of capsules [48], and both clonal stem fragments and buoyant sexual propagules are dispersed by water [13,54]. Molecular analyses within and among populations in California revealed limited genotypic and genetic variation, suggesting invasive spread has been primarily by hydrochorous dispersal of clonal propagules [54]. Yet, molecular results also provided evidence of sexual reproduction at a newly colonized restoration site where disturbance-generated gaps in the canopy were present [54]. Hydrochorous dispersal of fruits is likely, and seedlings can emerge from dehiscent capsules and raft in water [55]. *L. hexapetala* has a high potential for sexual recruitment and seed bank formation where conditions are favorable. Studies in France indicate *L. hexapetala* can produce 20 to 60 viable seeds per capsule and 10,000 seeds m^−2^ [55]. In *L. hexapetala* populations at the Laguna de Santa Rosa and Russian River (California), we typically found up to 80 seeds per capsule. Results of temperature response effects in growth chamber trials found >80% germination rates for *L. hexapetala* seeds from the Russian River and Laguna de Santa Rosa populations in California, and the germination capacity of *L. hexapetala* seeds was sustained under increased temperatures predicted from global warming models [56]. In outdoor common garden experiments conducted simultaneously in France and California, with reciprocal transplants of seeds from *L. hexapetala* populations from contrasting climates, the germination percentages and velocity increased or were maintained under warmer atmospheric and soil temperatures [32]. In addition, though survivorship of seedlings decreased with warming, the biomass of surviving seedlings increased in the warmer climate [56].

### 4.2. Seed Bank Sampling and Seedling Emergence Assay

In late winter (March), prior to spring emergence and growth of macrophytes, we established four field research sites in the Laguna de Santa Rosa. Using a space-for-time substitution, we established two study sites where invasive *L. hexapetala* had invaded and become dominant in the freshwater emergent plant community, including: Laguna Wildlife Area at Blucher Creek (LGBC; 38.377751°, −122.7833179°); Laguna Ranch near Santa Rosa Creek confluence with the Laguna channel (LGLR; 38.447897°, −122.835834°). We also established two additional study sites in nearby freshwater emergent wetlands within the same watershed, where *L. hexapetala* was not present, but that otherwise had similar environmental conditions to those of the invaded plots (e.g., proximity to stream channel; soil characteristics, as shown in Figure S3; presence of emergent wetland vegetation) (Laguna Wildlife Area near Gravenstein Creek (LGGC; 38.396287°, −122.807817°); Santa Rosa Creek upstream of Laguna confluence (SRCU; 38.459315, −122.654070)). At each wetland site, we randomly established and permanently marked five 5 × 5 m plots that were separated by a minimum of 10 m. From each plot, 10 replicate 4.8 cm diameter × 10 cm deep soil cores were collected for a total of 50 cores per site. We separated the upper 5 cm (surface) and the lower 5 cm (buried) half of each core sample. The samples were kept in the dark and refrigerated at +4 °C until processing. The samples were sieved, and coarse organic fragments, roots, and rhizomes were removed to eliminate potential recruitment from any buried asexual fragments; soil with seeds was retained. We then mixed 4 replicate surface soil cores from each of the two depth zones to create 4 composite surface seed banks and four composite buried seed banks per plot. The remaining replicate soil cores (also split by soil profile depth) from each plot were dried, weighed, and evaluated for bulk density, organic matter content by loss on ignition, and total C, N, and P. Eighty experimental seed banks were prepared by spreading composite sediment aliquot samples to a depth of 2 cm over sterilized sand in 2.8 l pots with drainage holes. No seedlings emerged from the samples prior to treatment establishment.

The seed bank was estimated using the seedling emergence method [37], as this method provides an accurate measure of viable seeds in wetland soil and the ability to assess the relationship between seed bank composition and field recruitment conditions [73,74]. Seedling emergence response to two treatment factors, hydrology (whole-plot factor) and sediment depth (between-plot factor), and their variation by wetland site (sub-plot factor) were tested in a 2 × 4 full factorial split plot arrangement with 5 replications. Treatment factors randomly assigned sediment aliquots from each of the four study sites including hydrology (+10 cm flooded; moist not flooded), and sediment depth (surface 0 to −5 cm depth; buried −5 to −10 cm depth). Hydrology (whole-plot factor) was randomly assigned to each of 20 80-l aquatic mesocosms in a glass house. Four individual experimental seed banks were then placed within each mesocosms according to the randomization scheme. De-ionized water was added to induce flood treatments (10 cm inundation above pot) and moist soil treatments (sub-irrigated, water level maintained −10 cm below pot surface) which were maintained throughout the experiments. Seed banks were initially examined for seedling emergence 5 times per week, followed by weekly census when emergence slowed for > 12 months (54 weeks), at which time seedling emergence ceased. Seedlings were either recorded and carefully removed after identification or transplanted and grown to maturity for taxonomic identification and to prevent the dispersal of mature seed into the experiment. Plants were identified on the basis of references [75,76]. The *Ludwigia* congeners are difficult to identify, particularly in pre-reproductive life stage, but taxa in the study area can be distinguished from one another by counting chromosome numbers [77,78]. Therefore, all *Ludwigia* seedlings that emerged were transplanted to flooded mesocosms to allow for the development of floating roots. Young root tips were then sampled from each, and somatic chromosome counts were determined to confirm species-level identification.

Total seedling emergence (number of seedlings per kg of soil sample) and species richness (number of taxa) of all emergent plants and of exotic taxa were analyzed as a split-plot experimental design by three-way analysis of variance ANOVA (SAS version 9.2 for Windows, SAS Institute, Cary, NC, USA) to detect differences in these factors across the four sites. Sediment depth and inundation were set as fixed qualitative factors with discrete categories. Total seedling emergence data were natural log (base e)-transformed to adjust for homogeneity of variances prior to ANOVA (Proc MIXED). Given the Poisson response distribution of plant taxa richness data, we used Proc GLIMMIX to fit the general linear mixed model. The number of exotic taxa responding to treatments was analyzed as both a Poisson distribution and a negative binomial response distribution, because of the assumption of large variance, using ANOVA (Proc GLIMMIX), which yielded very similar results. Therefore, given the larger variance, for the number of exotic taxa responding to treatments, we reported the negative binomial response to evaluate this factor (Proc GLIMMIX)

### 4.3. Standing Vegetation Assessment and Comparison to Seed Banks

We returned to the field plots at each study site during peak summer growth to measure vegetation development. We subdivided each 5 × 5 m plot into 1 m^2^ quadrats, recorded all plant taxa present, and determined the percent cover of each plant species. To examine the impact of *L. hexapetala* on invaded plant communities, we calculated mean species richness (number of discrete plant taxa), evenness (Shannon *E*), and Shannon H’ diversity [79] per plot for both invaded and non-invaded study sites. Differences in the abundance of *L. hexapetala* between invaded plots were tested with paired t-tests on arc-sign square root-transformed cover data; the resulting *p*-values were Bonferroni-adjusted. Sørensen’s coefficient of similarity [80] was used to compare floristic similarity among invaded and non-invaded wetlands.

## Figures and Tables

**Table 1:** Results of three-way ANOVA tests for the effect of wetland location (SITE), hydrology (HYD), soil profile depth (DPT) and their interactions on total number of seedlings, taxa richness of emergent seedlings, and number of exotic seedlings.

	Effect	Num DF	Den DF	F-Value	p
Total Seedlings	SITE	3	61	13.63	**<0.0001**
	HYD	1	61	145.26	**<0.0001**
	DPT	1	61	14.97	**0.0003**
	HYD*DPT	1	61	0	0.9769
	SITE*HYD	3	61	1.24	0.3022
	SITE*DPT	3	61	1.71	0.1743
	SITE*HYD*DPT	3	61	0.10	0.9600
Taxa Richness	SITE	3	64	7.35	**0.0003**
	HYD	1	64	198.95	**<0.0001**
	DPT	1	64	4.93	**0.0299**
	HYD*DPT	1	64	0.09	0.7609
	SITE*HYD	3	64	0.90	0.4480
	SITE*DPT	3	64	1.79	0.1587
	SITE*HYD*DPT	3	64	1.17	0.3275
Exotic Seedlings	SITE	3	64	15.76	**<0.0001**
	HYD	1	64	52.71	**<0.0001**
	DPT	1	64	9.33	**0.0033**
	HYD*DPT	1	64	0.02	0.8807
	SITE*HYD	3	64	0.96	0.4189
	SITE*DPT	3	64	3.53	**0.0196**
	SITE*HYD*DPT	3	64	1.59	0.2009

## Supplementary Materials

The following are available online at https://www.mdpi.com/2223-7747/8/11/451/s1, Table S1: Vascular plant taxa recorded as seed bank emergents and within extant vegetation at the Laguna de Santa Rosa; Table S2. Total counts of germinants, germinants per kg of soil core sample, total number of plant taxa, exotic and native germinants, and exotic and native plant taxa that emerged as seedlings from experimental seed banks under two hydrology treatments from soil seed banks collected at two sites invaded by *L. hexapetala*, compared to two nearby sites without *L. hexapetala*; Figure S3. Soil physico-chemical characteristics of sediment seed bank samples from the Laguna de Santa Rosa. Supplementary Data files: Data seed bank assay.xls, Data vegetation field plots_Diversity indices.xls, Data seed bank vs. vegetation_Sorenson indices.xls.
