# Supplementary material for: Seedling Emergence from Seed Banks in Ludwigia hexapetala-Invaded Wetlands: Implications for Restoration
Source: Plants (Basel). 2019 Oct 25;8(11):451. doi: 10.3390/plants8110451 (PMC6918176; doi:10.3390/plants8110451)

Seedling Emergence from Seed Banks in *Ludwigia hexapetala*-Invaded Wetlands: Implications for Restoration

Brenda J. Grewell, Morgane B. Gillard, Caryn J. Futrell, Jesús M. Castillo

Supplemental Materials

Table S1. Vascular plant taxa recorded as emergent seedlings from experimental seed banks (SB) and from extant vegetation (VEG) at the study sites (LGLR = Laguna at Laguna Ranch, LGBC = Laguna at Blucher Creek, LGGC = Laguna at Gravenstein Creek, SRCU = Santa Rosa Creek Upstream) in the Laguna de Santa Rosa Watershed. *Origin status of plants observed are indicated as E= exotic and N = native.

			INVADED				NOT INVADED			
			LGLR		LGBC		LGGC		SRCU	
**MONOCOT PLANT SPECIES**	**FAMILY**	**E/N***	**SB**	**VEG**	**SB**	**VEG**	**SB**	**VEG**	**SB**	**VEG**
Agrostis stolonifera	POACEAE	E			■		■	■	■	
Alisma lanceolatum	ALISMATACEAE	E	■	■	■		■	■	■	■
Alisma triviale	ALISMATACEAE	N					■			■
Bolboschoenus fluviatilis	CYPERACEAE	N					■			
Bolboschoenus maritimus	CYPERACEAE	N	■						■	
Callitriche heterophylla	CALLITRICHACEAE	N					■			
Crypsis schoenoides	POACEAE	E	■	■	■		■	■	■	■
Cyperus difformis	CYPERACEAE	E	■				■			
Cyperus eragrostis	CYPERACEAE	N	■	■	■	■	■	■	■	■
Cyperus erythrorhizos	CYPERACEAE	N	■	■	■		■		■	
Eleocharis acicularis	CYPERACEAE	N	■	■	■		■		■	■
Eleocharis macrostachya	CYPERACEAE	N	■		■		■	■	■	■
Glyceria occidentalis	POACEAE	N					■			■
Isolepis cernua	CYPERACEAE	N							■	
Juncus bufonius	JUNCACEAE	N	■	■	■				■	■
Juncus effusus	JUNCACEAE	N								■
Juncus oxymeris	JUNCACEAE	N							■	■
Juncus phaeocephalus	JUNCACEAE	N					■	■		
Juncus xiphioides	JUNCACEAE	N					■		■	■
Leptochloa fusca ssp. fascicularis	POACEAE	N	■						■	
Leersia orzoides	POACEAE	N							■	■
Lolium multiflorum	POACEAE	E	■					■		
Paspalum distichum	POACEAE	N	■	■	■		■	■	■	■
Phalaris aquatica	POACEAE	E	■							■

Phalaris arundinacea	POACEAE	N								
Polypogon monspeliensis	POACEAE	E								
Scoenoplectus acutus	CYPERACEAE	N								
Schoenoplectus americanus	CYPERACEAE	N								
Schoenoplectus californicus	CYPERACEAE	N								
Scirpus microcarpus	CYPERACEAE	N								
Sparganium eurycarpum	TYPHACEAE	N								
Typha angustifolia	TYPHACEAE	N								
Typha latifolia	TYPHACEAE	N								

			INVADED				NOT INVADED			
			LGLR		LBBC		LGGC		SRCU	
**DICOT PLANT SPECIES**	**FAMILY**	**E/N***	**SB**	**VEG**	**SB**	**VEG**	**SB**	**VEG**	**SB**	**VEG**
Ammania robusta	LYTHRACEAE	N								
Atriplex prostrata	CHENOPODIACEAE	E								
Bidens frondosa	ASTERACEAE	N								
Bidens laevis	ASTERACEAE	N								
Chamaesyce serpyllifolia	EUPHORBIACEAE	N								
Convolvulus arvensis	CONVULVULACEAE	E								
Conyza canadensis	ASTERACEAE	N								
Crassula aquatica	CRASSULACEAE	N								
Cuscuta indecora	CUSCUTACEAE	N								
Cypselea humifusa	AIZOACEAE	E								
Epilobium ciliatum ssp. ciliatum	ONAGRACEAE	N								
Epilobium densiflora	ONAGRCEAE	N								
Eryngium aristulatum	APIACEAE	N								
Gnapthalium palustre	ASTERACEAE	N								
Lactuca serriola	ASTERACEAE	E								
Lepidium latifolium	BRASSICACEAE	E								
Lotus corniculatus	FABACEAE	E								
Ludwigia hexapetala	ONAGARACEAE	E								
Ludwigia palustris	ONAGARACEAE	N								
Ludwigia peploides ssp montevidensis	ONAGARACEAE	E								
Ludwigia peploides ssp peploides	ONAGARACEAE	E								
Lythrum hyssopifolia	LYTHRACEAE	E								
Lythurum salicaria	LYTHRACEAE	E								
Melilotus alba	FABACEAE	E								
Mentha pulegium	LAMIACEAE	E								
Mimulus guttatus	PHRYMACEAE	N								
Montia fontana	PORTULACACEAE	N								
Montia linearis	PORTULACACEAE	N								
Myriophyllum aquaticum	HALORAGACEAE	E								
Phyla nodiflora	VERBENACEAE	N								
Helminthotheca echioides (x-Picris)	ASTERACEAE	E								
Plantago subnuda	PLANTAGINACEAE	N								

Persicaria amphibia (x-Polygonum)	POLYGONACEAE	N
Persicaria hydropiperoides	POLYGONACEAE	N
Persicaria lapathifolia (xPolygonum)	POLYGONACEAE	N
Persicaria pensylvanica	POLYGONACEAE	E
Persicaria punctata	POLYGONACEAE	N
Polygonum aviculare ssp. depressum	POLYGONACEAE	E
Rorippa curvisiliqua	BRASSICACEAE	N
Nasturtium officinale	BRASSICACEAE	N
Raphansus sativa	BRASSICACEAE	E
Rorripa nasturtium-aquaticum	BRASSICACEAE	N
Rorripa palustris	BRASSICACEAE	N
Rumex crispus	POLYGONACEAE	E
Salix exigua	SALICACEAE	N
Sonchus oleraceus	ASTERACEAE	E
Spergularia rubra	CARYOPHYLLACEAE	E
Stachys albens	LAMIACEAE	N
Symphyotrichum subulatum	ASTERACEAE	N
Urtica dioica	URTICACEAE	N
Veronica americana	PLANTAGINACEAE	N
Veronica anagallis-aquatica	PLANTAGINACEAE	E
Veronica catenata	PLANTAGINACEAE	E
Veronica peregrina ssp. xalapensis	PLANTAGINACEAE	N
Xanthium strumarium	ASTERACEAE	N

Table S2. Total counts of germinants, germinants per kg of soil core sample, total number of plant taxa, exotic and native germinants, and exotic and native plant taxa that emerged as seedlings from experimental seed banks under two hydrology treatments (F = flood inundate; D = Drawdown, Sub-irrigated) from soil seed banks collected at two sites invaded by *L. hexapetala* (LGLR = Laguna de Santa Rosa at Laguna Ranch; LGBC = Laguna de Santa Rosa at Blucher Creek confluence) compared to two nearby sites without *L. hexapetala* (Santa Rosa Creek upstream of Laguna de Santa Rosa confluence; LGGC = Laguna de Santa Rosa at Gravenstein Creek confluence).

TREATMENT				RESPONSE COUNTS						
SITE	HYD	DEP	REP	Total Germinants	Germinants (per kg soil)	TAXA	Exotic Germinants	Native Germinants	Exotic Taxa	Native Taxa
SRCU	F	U	1	23	63.2	5	9	14	1	4
SRCU	F	U	2	10	27.5	3	3	7	1	2
SRCU	F	U	3	5	13.7	2	4	1	1	1
SRCU	F	U	4	7	19.2	2	5	2	1	1
SRCU	F	U	5	5	13.7	2	4	1	1	1
SRCU	F	L	1	4	9.7	3	0	4	0	3
SRCU	F	L	2	2	4.9	1	0	2	0	1
SRCU	F	L	3	4	9.7	1	4	0	1	0
SRCU	F	L	4	0	0.0	0	0	0	0	0
SRCU	F	L	5	0	0.0	0	0	0	0	0
SRCU	D	U	1	184	505.5	20	19	165	7	13
SRCU	D	U	2	41	112.6	9	2	39	2	7
SRCU	D	U	3	64	175.8	16	3	61	2	14
SRCU	D	U	4	33	90.7	11	10	23	2	9
SRCU	D	U	5	38	104.4	13	16	22	4	9
SRCU	D	L	1	65	157.8	17	15	50	5	12
SRCU	D	L	2	23	55.8	12	1	22	1	11
SRCU	D	L	3	5	12.1	4	0	5	0	4
SRCU	D	L	4	31	75.2	6	0	31	0	6
SRCU	D	L	5	20	48.5	7	6	14	3	4
LGGC	F	U	1	16	55.4	3	4	12	2	1
LGGC	F	U	2	47	162.6	4	35	12	2	2
LGGC	F	U	3	22	76.1	4	7	15	2	2
LGGC	F	U	4	32	110.7	3	11	21	2	1
LGGC	F	U	5	34	117.6	3	17	17	1	2
LGGC	F	L	1	12	30.8	4	5	7	2	2
LGGC	F	L	2	10	25.7	6	3	7	1	5
LGGC	F	L	3	15	38.6	5	2	13	1	4
LGGC	F	L	4	12	30.8	4	5	7	2	2
LGGC	F	L	5	39	100.3	3	14	25	1	2
LGGC	D	U	1	259	896.2	17	43	216	5	12
LGGC	D	U	2	201	695.5	21	108	93	7	14
LGGC	D	U	3	96	332.2	20	47	49	7	13
LGGC	D	U	4	107	370.2	15	31	76	6	9
LGGC	D	U	5	128	442.9	14	80	48	6	8
LGGC	D	L	1	46	118.3	11	18	28	3	8
LGGC	D	L	2	54	138.8	13	18	36	4	9
LGGC	D	L	3	97	249.4	17	48	49	8	9
LGGC	D	L	4	103	264.8	14	27	76	3	11
LGGC	D	L	5	123	316.2	14	41	82	7	7
LGBC	F	U	1	11	35.7	3	2	9	0	3
LGBC	F	U	2	11	35.7	3	5	6	0	3
LGBC	F	U	3	0	0.0	0	0	0	0	0

LGBC	F	U	4	1	3.2	1	0	1	0	1
LGBC	F	U	5	6	19.5	2	5	1	1	1
LGBC	F	L	1	23	67.8	3	22	1	2	1
LGBC	F	L	2	9	26.5	1	9	0	1	0
LGBC	F	L	3	1	2.9	1	0	1	0	1
LGBC	F	L	4	5	14.7	2	2	3	1	1
LGBC	F	L	5	8	23.6	2	6	2	1	1
LGBC	D	U	1	38	123.4	7	13	25	3	4
LGBC	D	U	2	98	318.2	18	36	62	7	11
LGBC	D	U	3	38	123.4	7	3	35	2	5
LGBC	D	U	4	61	198.1	11	13	48	4	7
LGBC	D	U	5	33	107.1	8	5	28	2	6
LGBC	D	L	1	84	247.8	9	18	66	2	7
LGBC	D	L	2	41	120.9	5	8	33	3	2
LGBC	D	L	3	11	32.4	6	2	9	2	4
LGBC	D	L	4	119	351.0	14	11	108	5	9
LGBC	D	L	5	36	106.2	12	15	21	5	7
LGLR	F	U	1	12	41.4	5	6	6	2	3
LGLR	F	U	2	12	41.4	4	9	3	2	2
LGLR	F	U	3	3	10.3	3	1	2	1	2
LGLR	F	U	4	20	69.0	4	15	5	1	3
LGLR	F	U	5	19	65.5	2	18	1	1	1
LGLR	F	L	1	4	14.3	3	1	3	1	2
LGLR	F	L	2	3	10.7	2	2	1	1	1
LGLR	F	L	3	4	14.3	2	4	0	2	0
LGLR	F	L	4	7	25.0	2	6	1	1	1
LGLR	F	L	5	14	50.0	3	12	2	1	2
LGLR	D	U	1	189	651.7	17	62	127	7	10
LGLR	D	U	2	442	1524.1	28	225	217	12	16
LGLR	D	U	3	72	248.3	10	7	65	2	8
LGLR	D	U	4	82	282.8	13	15	67	4	9
LGLR	D	U	5	54	186.2	10	16	38	3	7
LGLR	D	L	1	134	478.6	16	36	98	6	10
LGLR	D	L	2	205	732.1	19	71	134	7	12
LGLR	D	L	3	41	146.4	11	13	28	7	4
LGLR	D	L	4	31	110.7	7	8	23	3	4
LGLR	D	L	5	76	271.4	14	32	44	4	10

Figure S3. Soil physico-chemical characteristics of surface (U) and buried (L) sediment seed bank samples from the Laguna de Santa Rosa. Sites codes for uninvaded sites are: Laguna at Gravenstein Creek (LGGC); Santa Rosa Creek above Laguna (SRCU). Site codes for *L. hexapetala* invaded sites are: Laguna at Laguna Ranch (LGLR); Laguna at Blucher Creek (LGBC). Data points represent means with 95% confidence intervals. Some error bars are too small to be visible.

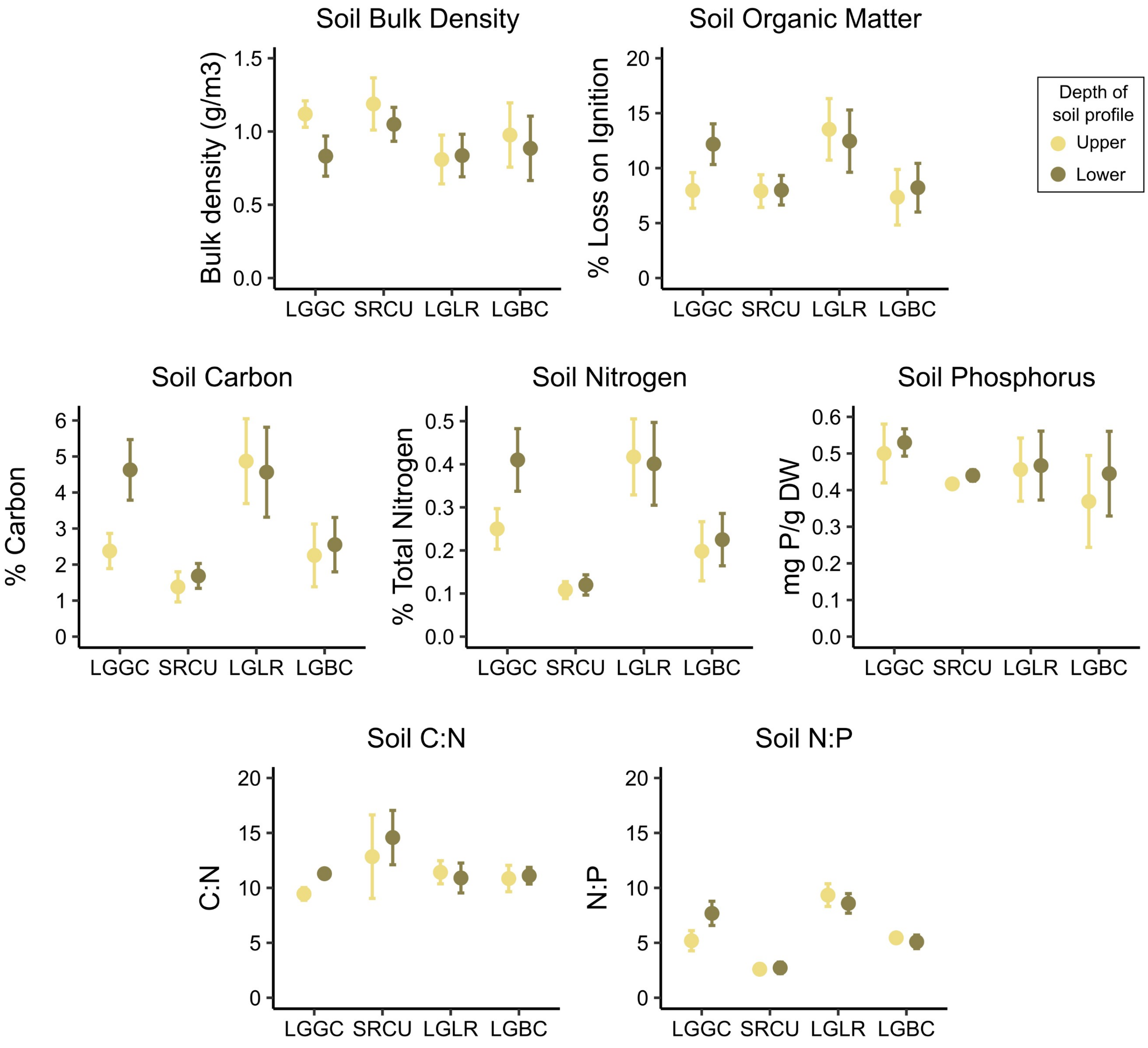

Supplement: Supplementary file 1 [file plants-08-00451-s001.pdf]
